# Determining the weighting and relative importance of CanMEDS roles and competencies

**DOI:** 10.1186/1756-0500-5-354

**Published:** 2012-07-16

**Authors:** Brenda J Stutsky, Marilyn Singer, Robert Renaud

**Affiliations:** 1 Faculty of Medicine, Division of Continuing Professional Development, University of Manitoba, Winnipeg, MB, Canada; 2 Faculty of Education, Department of Educational Administration, Foundations, and Psychology, University of Manitoba, Winnipeg, MB, Canada

**Keywords:** CanMEDS, Weighting, Practice assessment, Competency assessment, Competencies, Physician roles

## Abstract

**Background:**

The CanMEDS roles and competencies are being used as the framework to support the development of the Manitoba Practice Assessment Program (MPAP) designed to assess the competence of physicians practicing with a conditional license. Establishing the link between clinical practice and assessment of performance is critical in the development of the MPAP. A first step in establishing this link is to identify activities performed in actual clinical practice as well as the importance of those activities.

**Methods:**

A descriptive survey design was used to answer the research questions: (1) How do physicians rate the complexity, frequency, and criticality of CanMEDS roles? (2) What is the distribution of perceived importance scores for the CanMEDS roles? Two online surveys, one specific to family practice physicians, and one specific to specialists, were emailed to a sample of Canadian physicians.

**Results:**

Overall perceived importance scores were calculated for each of the CanMEDS roles. It appears that each role is considered to be at least moderately important. The Medical Expert role was ranked as the most important, followed by the roles of Communicator, Professional, Collaborator, Scholar, Manager, and Health Advocate. There were no significant differences in overall CanMEDS perceived importance scores between family practice physicians and specialists (*N* = 88).

**Conclusions:**

Given that each of the CanMEDS roles is considered at least moderately important, a variety of assessment tools are needed to evaluate competencies across the entire spectrum of roles. The results underscore the importance of incorporating a multifaceted approach when developing a practice assessment program.

## Background

The University of Manitoba, Faculty of Medicine, Division of Continuing Professional Development is embarking on the development of an assessment program for practicing physicians called the Manitoba Practice Assessment Program (MPAP). The MPAP is being developed in response to a need identified by the College of Physicians and Surgeons of Manitoba (CPSM). Within the Province of Manitoba, physicians commonly enter practice on a conditional license. A conditional license allows physicians who have completed post-graduate training, but have not yet achieved their Canadian certification through The College of Family Physicians of Canada (CFPC) or the Royal College of Physicians and Surgeons of Canada (RCPSC), to enter supervised practice within a defined time frame during which they are expected to complete the appropriate certification exam. There are a significant number of physicians who are in established practices and have not yet achieved final certification for a variety of reasons. The CPSM requested that the University of Manitoba, Faculty of Medicine develop a process to assess such physicians in their practice settings to enable the CPSM to make a decision about granting registration to these candidates.

The framework for the development of the MPAP is the Canadian Medical Education Directions for Specialists (CanMEDS) physician competency framework. The establishment of the CanMEDS framework is rooted in medical education with the framework originally developed to prepare physicians to thrive in our ever-changing healthcare environment [1]. Since 1993, the framework has evolved through four major phases with the current 2005 version highlighting the seven core roles of a physician: Medical Expert, Communicator, Collaborator, Manager, Health Advocate, Scholar, and Professional [1]. Key and enabling competencies have been developed for each of the core CanMEDS roles. A trademarked diagram, known as either the CanMEDS *cloverleafdaisy*, or *flower*, was produced by the RCPSC to visually illustrate the core roles and interconnections among the roles [1]. Essentially, the Medical Expert is in the middle of the daisy representing the central role, with each of the remaining roles represented by an overlapping petal that is equal in size to one another.

The CFPC [2] adopted the CanMEDS framework developed by the RCPSC [3]; however, it was customized to reflect the specific competencies of family practice physicians specifically in the Medical Expert role with the addition of two competencies, and the Collaborator and Professional roles with one competency being added to each role. Other than these additions and a few minor wording changes, the two frameworks are essentially the same. The RCPSC specialties and sub-specialties have also adapted the CanMEDS framework to reflect their specific practices [4].

The CanMEDS framework has been incorporated into undergraduate and postgraduate educational programs both within Canada and internationally [5-11]. Successes can be attributed to a detailed implementation strategy that has focused on four key areas: (1) standards for curriculum, teaching, and assessment; (2) faculty development; (3) research and development resources; and (4) outreach and communications [12]. Gaps, however, have been noted between the ideal delivery of medical education that incorporates all of the core CanMEDS roles into the curriculum as opposed to a focus on the role of Medical Expert [13,14]. Frank and Danoff [12] acknowledge that resistance to change, faculty overload, and scarce financial resources are challenges that limit the adoption of the CanMEDS framework.

The concept of using the CanMEDS framework as a foundation for an assessment program for practicing physicians is relatively novel as the focus has been on undergraduate, postgraduate, and continuing medical education. Researchers have started to examine the importance rankings of the CanMEDS competencies; however, the context of the research focused on the applicability of the CanMEDS roles and competencies for international physicians [8,9]. To date, no studies have been located in which researchers specifically examined the activity weight scores of roles by asking practicing Canadian physicians to rate the criticality or consequence of performing competencies key to their practice incorrectly or not at all, or by rating the frequency in which they perform these key competencies on an everyday basis.

It is important that a credentialing or licensing program be able to provide empirical evidence of the link between the knowledge, skills, and attitudes tested in an examination and the activities performed in practice [15]. Although the MPAP is not a credentialing or licensing program, the MPAP assessment process is considered a high-stakes assessment given that the outcome of the assessment will contribute to the CPSM’s decision regarding registration. Establishing the link between clinical practice and assessment of performance is critical in the development of the MPAP, and a first step in establishing this link is to clearly identify activities performed in actual clinical practice, for assessment tools need to include all roles and competencies and not just focus on the Medical Expert role. In addition, the weighting of the various roles needs to be determined and taken into consideration when developing the assessment tools and determining the final pass/fail outcome of an assessment.

The goal of the study was to determine the importance of physician’s roles and competencies as perceived by a sample of Canadian physicians. The following questions guided the research:

1. How do physicians rate the complexity, frequency, and criticality of CanMEDS roles?

2. What is the distribution of perceived importance scores for the CanMEDS roles?

## Methods

A descriptive survey design was used to answer the research questions and achieve the study goal by asking practicing physicians to rate competencies identified as key to their practice. Ultimately, the results will be taken into consideration in the development of the assessment tools and processes that will be used by the MPAP in assessing the competence of practicing physicians.

### Procedure

After receiving ethical approval from the University of Manitoba Bannatyne Campus Research Ethics Boards (File Number: H2010:357) to conduct the study, a list of Canadian physicians residing in British Columbia, Alberta, Saskatchewan, Manitoba, North West Territories, Yukon Territory, and Nunavut was generated by the principal investigator from the online database called, *Scott’s Directories: Canadian Medical Directory* via a departmental username and password. Information obtained from the directory included the physician’s first and last name, province of residence, and email address. Physicians that met the inclusion criteria, which was a valid email address, were included.

Due to the large participant list, and to avoid the recruitment notice being identified as unsolicited mail, an email distribution service, namely, *Constant Contact*, was used to send the electronic recruitment notice. Included in the recruitment notice was general participant information, an electronic copy of the consent form, and links to two online surveys. The principal investigator designed and sent the recruitment email to potential participants. If an email was returned undelivered, no attempt was made to resend the email. Physicians were solely responsible for reading the participant information and consent form and determining for themselves whether to participate in the study. Participants were not required to sign and return the informed consent, and completion of the online survey constituted consent to participate. In addition, a statement of consent was included on the introductory page of the online survey. Participants were provided with contact information for all of the investigators as well as the university research ethics board coordinator if they had any questions, issues, or concerns.

After deciding to participate, physicians completed one of two researcher developed online surveys accessible via an encrypted subscription of *Survey Monkey*. Physicians with a clinical background in Family Practice were asked to complete the survey entitled, *MPAP: CanMEDS Competencies for Family Practice.* Physicians with a Specialty Practice clinical background were asked to complete the survey entitled, *MPAP: CanMEDS Competencies for Specialty Practice*. A period of two months was given to complete the survey. One month after sending out the initial recruitment email, the principal investigator sent a follow-up reminder email to all participants. Since it was not known who completed the surveys, all participants received the follow-up reminder email. Constant Contact has an option for opting out of the email service, and those physicians that opted out of receiving any further emails after the initial recruitment email did not receive the follow-up email. Approximately one month after access to the surveys closed, overall results were sent to all participants who received the initial recruitment email and had not opted out of receiving future emails.

### Surveys

**Figure 1:**
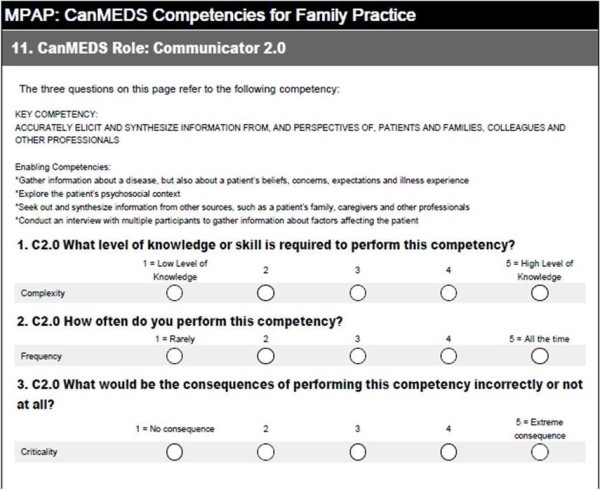
Example of survey questions.

The surveys were developed by the investigators applying the work of Raymond [15] and using the actual wording of CanMEDS roles, key competencies, and enabling competencies from the RCPSC [3] and the CFPC [2]. Based on a table of scales for rating practice activities and practice-related responsibilities from a variety of studies [15], the following questions were developed to measure complexity, frequency, and criticality of each of the key competencies: (1) Complexity: What level of knowledge or skill is required to perform this competency? (2) Frequency: How often do you perform this competency? (3) Criticality: What would be the consequences of performing this competency incorrectly or not at all? A rating scale of 1 to 5 was used for all three questions (see Figure 1). Family practice physicians were asked to score 31 key CFPC competencies, and specialty practice physicians were asked to score 28 key RCPSC competencies based on the frequency, complexity, and criticality of the competency in their own practice. Included in the survey was a demographic section where physicians described their practices as active or inactive, and urban, rural, or northern/remote. Time to complete the survey was approximately 30 minutes.

### Data Analysis

An overall index of perceived importance for each competency (i) within its respective CanMEDS role was calculated using a multiplicative model

(1) $$I_{i}=F_{i}C_{i}$$

where *I*_*i*_ = overall importance, *F*_*i*_ = frequency, and *C*_*i*_ = criticality [15-17]. A common limitation of the multiplicative model occurs when the range of criticality ratings is relatively narrow compared to the variability of frequency ratings leading to biased ratings of overall importance. In this instance, it is necessary to transform the criticality ratings to equalize their contributions in calculating perceived overall importance [16]. However, in our data, because these descriptives for each competency were not markedly different, the variables were not transformed. Moreover, the pattern of ratings derived from the multiplicative model are comparable to other complex methods of combining frequency and criticality ratings [17]. An index of practice importance for each role was obtained by calculating the mean perceived overall importance of the competencies within the role [15].

## Results and discussion

A total of 3,294 participants meeting the study criteria received the initial recruitment email. Five hundred and sixty two emails were returned undelivered, and a total of 27 participants opted out of receiving emails from the research team over the course of the study. Thirty five participants accessed and started to complete the MPAP: CanMEDS Competencies for Family Practice survey and 92 participants accessed and started to complete the MPAP: CanMEDS Competencies for Specialty Practice survey. Scores from those participants who did not complete the majority of survey questions were deleted prior to analysis of data. The final sample (*N* = 88) consisted of 23 participants completing the family practice survey and 65 completing the specialty practice survey. Although the 3% response rate fell well short of an initial expected minimum of 10%, based on the work of Tannenbaum and Wesley [18] who concluded that subject matter experts (N = 7) and field respondents (N = 329 and 423) provided similar ratings of importance for knowledge and ability domains in job analysis surveys, the results do provide promising preliminary data upon which further studies can build. Numerous factors may have contributed to the low response rate including the length of the survey that included 95 questions for family practice physicians and 86 questions for specialty practice physicians. Physicians may not have had the time to complete such a lengthy survey without any compensation or tangible incentive for participation. The fact that the survey was an online survey may have contributed to the low response rate, for according to the Constant Contact statistics, 517 potential respondents did not even open the email request to participate, and as noted, 562 emails were not delivered due to invalid email addresses. In hindsight, using a marketing service database to identify potential research participants (i.e., Scott’s Directories: Canadian Medical Directory) may not have been optimal, for those physicians on the list likely get numerous unwanted emails and the request to participant may have been perceived as *junk* mail. Strategies to increase the response rate may include targeting specific physician groups through physician organizations and special interest groups, as well as distributing hardcopy surveys as opposed to using an online survey; however, hardcopy distribution will add to the cost of the survey in terms of the mail-out itself and data entry costs. A follow-up focus group to review the results may also be helpful in the validation process. Despite the low response rate, the researchers are unaware of any other work being done to attempt to weight the CanMEDS roles; therefore, the results are an important step in the continuous validation of the CanMEDS framework. Of those physicians that participated, 70 claimed to be in active practice, three referred to themselves as inactive practice, and 15 did not declare their status. Location of participants’ practice was noted as urban (n = 52), rural (n = 3), or northern/remote (n = 1), while 7 noted a combination of locations, and 25 did not note a practice location.

The mean ratings of complexity, frequency, criticality, and perceived overall importance for the competencies in each role are shown in Table 1. For complexity, the roles were fairly consistent, ranging from 3.81 (Professional) to 4.37 (Medical Expert) indicating that most competencies within each role required a moderate to high level of skill. The mean perceived frequencies were slightly more varied, ranging from 3.32 (Health Advocate) to 4.22 (Medical Expert). The Medical Expert role had the highest mean rating of Criticality at 4.09 compared to the least critical role, namely Health Advocate at 3.41. As mentioned earlier, perceived overall importance represented the product of the mean scores of frequency and criticality. As such, the lowest and highest possible mean scores of perceived overall importance range from 1 to 25 respectively. In sum, it appears that each role is considered to be at least moderately important ranging from 11.93 (Health Advocate) to 17.71 (Medical Expert). No significant differences in mean perceived importance scores were found in any of the CanMEDS roles between those participants that completed the family practice survey and those that completed the specialty practice survey (see Table 2).

**Table 1:** Descriptives for CanMEDS Roles

**CanMEDS Role**	***n***	**Complexity** ***M*** **(** ***SD*** **)**	**Frequency** ***M*** **(** ***SD*** **)**	**Criticality** ***M*** **(** ***SD*** **)**	**Overall Importance** ***M*** **(** ***SD*** **)**
Medical Expert	88	4.37 (0.47)	4.22 (0.71)	4.09 (0.54)	17.71 (4.08)
Communicator	88	4.03 (0.71)	4.20 (0.79)	3.82 (0.66)	16.42 (4.50)
Collaborator	86	3.99 (0.87)	3.86 (0.85)	3.69 (0.76)	14.56 (4.82)
Manager	85	3.97 (0.76)	3.57 (0.88)	3.42 (0.73)	12.72 (4.65)
Health Advocate	84	3.99 (0.77)	3.32 (0.94)	3.41 (0.73)	11.93 (4.66)
Scholar	84	4.20 (0.72)	3.67 (0.92)	3.51 (0.70)	13.30 (4.75)
Professional	83	3.81 (0.86)	3.86 (0.75)	3.86 (0.72)	15.31 (4.51)

**Table 2:** Comparison of mean importance scores for family practice and specialty physicians

**CanMEDS Role**		***n***	***M***	***SD***	***t***	***P***
Medical Expert	Family Specialty	23 65	17.07 17.93	3.69 4.21	-.87	.38
Communicator	Family Specialty	23 65	17.29 16.11	2.81 4.94	1.08	.28
Collaborator	Family Specialty	23 63	15.04 14.40	3.81 5.16	.55	.59
Manager	Family Specialty	22 63	12.52 12.79	4.35 4.79	-.24	.82
Health Advocate	Family Specialty	22 62	12.30 11.08	3.95 4.90	.43	.67
Scholar	Family Specialty	22 62	12.20 13.69	4.13 4.93	−1.27	.21
Professional	Family Specialty	22 61	16.22 14.98	3.78 4.73	1.11	.27

The finding that there was no significant difference in perceived overall importance scores for CanMEDS roles between participants that completed the family practice survey and the specialty practice survey is an important result as the research team moves forward in the development of a physician practice assessment program. The finding supports the notion that one set of comprehensive practice assessment tools can be developed to assess both family practice and specialty practice physicians using the CanMEDS roles and competencies as the underlying framework; however, additional specialty specific competencies will need to be included in specialty specific assessment tools.

The findings support the work of Frank [1] in that the Medical Expert role, that received the highest perceived overall importance score, is a central integrative role embodied by competent physicians. However, when examining the holistic practice of physicians, the roles of Communicator, Collaborator, Health Advocate, Manager, Scholar, and Professional play an important part in supporting the role of Medical Expert. The perceived importance score of the Collaborator role was somewhat surprising, especially given the focus on interprofessional practice and the need to engage with other physicians and health professionals on a daily basis in order to provide quality patient care. Specific reference to communities and the population at large, in questions pertaining to the Health Advocate role, may have contributed to the low ranking by specialty physicians in particular, for time constraints in their practices may not allow for health advocacy at this more global level. Possibly a larger sample size would have yielded different results.

The importance scores of each of the CanMEDS roles will be beneficial in determining a physician’s overall competence as assessed in a practice assessment program. It is expected that the relative importance of each of the roles will be taken into consideration when determining an overall pass or fail grade. Beyond assessment, importance and complexity scores can be used as a reference when developing undergraduate, postgraduate, and continuing medical education curricula.

Interestingly, Frank [1] notes that based on history and tradition, the CanMEDS roles are ranked as follows: Medical Expert, Communicator, Collaborator, Manager, Health Advocate, Scholar, and Professional. Given the relative importance ranking of roles from this study, and the fact that Whitehead, Austin, and Hodges [19] question the historical ranking of the roles, it may be that the ordering of the CanMEDS roles will need to change with further validation of results?

As the CanMEDS roles and competencies continue to be adopted in all facets of medicine, it is important that the framework continues to be validated. The small sample size limits the generalizability of the findings and researchers need to continue to examine perceived importance scores of the CanMEDS roles with larger samples and with specific specialties and sub-specialties. Many specialty groups certified by the RCPSC, for example, have adapted the CanMEDS roles and competencies to their own practices [4], and similar to the finding of Ringsted et al. [9] that discovered differences in importance ratings among specialties, it would be expected that the importance scores from one specialty to the other may be different. For example, the importance score for the Communicator role would be expected to be different for a psychiatrist compared to a pathologist. The non-differentiation of sub-specialties is a limitation of this study.

## Conclusions

Given that each of the CanMEDS roles is considered at least moderately important, it is assumed that a variety of assessment tools are needed to evaluate competencies across the entire spectrum as opposed to focusing on the role of Medical Expert. Overall, the results underscore the importance of incorporating a multifaceted approach when developing a practice assessment program.

## Abbreviations

CanMEDS, Canadian Medical Education Directions for Specialists; CFPC, The College of Family Physicians of Canada; CPSM, College of Physicians and Surgeons of Manitoba; MPAP, Manitoba Practice Assessment Program; RCPS, Royal College of Physicians and Surgeons of Canada.

## Competing interests

The authors declare that they have no competing interests.

## Authors’ contributions

BJS conceived of the study, lead all aspects of the study, and drafted the manuscript. MS participated in the design of the study and surveys. RR performed the statistical analyses and drafted related sections in the paper. All authors read, edited, and approved the final manuscript.

## Authors’ information

BS: RN, BN, MScN, EdS, PhD University of Manitoba, Faculty of Medicine, Division of Continuing Professional Development, Program Advisor Manitoba Practice Assessment Program, Director of e-Learning

MS:MD, FCFP University of Manitoba, Faculty of Medicine, Division of Continuing Professional Development Director, Clinician Assessment Programs

RR: PhD University of Manitoba, Faculty of Education Department of Educational Administration, Foundations, and Psychology Associate Professor
